# HIV-1-related factors interact with p53 to influence cellular processes

**DOI:** 10.1186/s12981-023-00563-7

**Published:** 2023-09-10

**Authors:** Shanling Liu, Ting Guo, Jinwei Hu, Weiliang Huang, Pengfei She, Yong Wu

**Affiliations:** 1 Department of Laboratory Medicine, The First Hospital of Changsha, 311 Yingpan Road, Changsha, 410005 Hunan China; 2 Department of Laboratory Medicine, Third Xiangya Hospital, Central South University, Changsha, 410013 Hunan China

**Keywords:** HIV-1, p53, Regulation, Molecular mechanism

## Abstract

Human immunodeficiency virus type 1 (HIV-1) is the primary epidemic strain in China. Its genome contains two regulatory genes (*tat* and *rev*), three structural genes (*gag*, *pol*, and *env*), and four accessory genes (*nef, vpr, vpu*, and *vif*). Long terminal repeats (LTRs) in thegenome regulate integration, duplication, and expression of viral gene. The permissibility of HIV-1 infection hinges on the host cell cycle status. HIV-1 replicates by exploiting various cellular processes via upregulation or downregulation of specific cellular proteins that also control viral pathogenesis. For example, HIV-1 regulates the life cycle of p53, which in turn contributes significantly to HIV-1 pathogenesis. In this article, we review the interaction between HIV-1-associated factors and p53, providing information on their regulatory and molecular mechanisms, hinting possible directions for further research.

## Background

Human immunodeficiency virus type 1 (HIV-1) is highly contagious to the active macrophages and proliferative CD4^+^ T lymphocytes [1]. Its genome comprises two identical positive-strand RNA, totaling 9.7 kilobase pairs (kbp) wrapped in a viral protein shell. The nucleocapsid is encircled by a phospholipid bilayer originated from the host cytomembrane, which contains virus-encoded membrane proteins. The genome regulates viral infectivity and pathogenesis, while the *gag* gene encodes a nucleocapsid protein. Two glycoproteins are encoded by *env* gene, namely, the envelope glycoproteins Gp120 and Gp41, that are required for viral infection. The *pol* gene encodes the key enzymes of viral replication, such as protease, conjugase, and reverse transcriptase. As the reverse transcriptase does not have a correction function, the replication frequency of the virus in vivo is high. Gene recombination occurs between the viral and host DNA. It is prone to random mutations and leads to drug resistance. Therefore, HIV-1 is highly variable. The frequency of variations in each part of the genome differs, with the *env* gene being the most susceptible to such variations [2–4].

Since its discovery many years ago, protein 53 (p53) has been a transcription factor encoded by the tumor protein 53 (TP53) gene in humans [5]. p53 has been an essential anti-tumor factor, and its response can be activated by various stress signals, including ribosome stress, activation of oncogene, stress of genetic toxicity, fluctuation in trophic, and oxygen deficit. p53 activates or suppresses the expression of several genes participated in cancer development [6]. Its inactivation facilitates the development of several human cancers and can be caused by different events, such as mutations in the *p53* gene (with or without deletion of related alleles) or combination of cellular or viral proteins, such as the human papillomavirus (HPV) E6 oncoprotein, to the *p53* gene [7]. Mutations in *p53* most often happen to exon 58, which is a highly conservative DNA-binding domain region. The p53 protein regulate its subcellular localization, stabilization, and conformation after undergoing extensive post-translational modifications [8]. Although p53 exists at low levels under physiological status, it is quickly activated and stabilized by a series of stimulations, such as compounds that cause DNA damage [9]. Activated p53 participates in various physical processes, including apoptosis induction through transcription-dependent and-independent mechanisms, as well as the induction of cell cycle arrest at the G1/S and G2/M checkpoints. Some well-known transcriptional targets of p53 are p21/waf1, MDM2, 14-3-3, p53-R2, GADD45, FAS, Killer/DR5, IGF-BP3, AIP1, and PIG3, which participate in cell cycle control, DNA repair regulation, differentiation, aging, and control of p53 activity and stability [10, 11]. HIV-1 replicate by exploiting various cellular processes via upregulation or downregulation of specific cellular proteins that regulate viral pathogenesis. Proteins encoded by HIV-1 interact with p53 and regulate its function. p53 and its downstream gene *p21* disturb the early replicative stage of HIV-1 in non-circulating cells and human monocyte derived macrophages (hMDMs) [12]. *p53* gene is thought to be crucial in inhibiting HIV-1 infection. Moreover, HIV-1 regulates the life cycle of p53 while p53 contributes significantly to its pathogenesis.

The regulation between HIV-1-related factors and p53 plays important roles in cell cycle blockage, apoptosis, HIV-1 virus replication, and other cellular processes. Here, we review the literature to gain insights into the importance and molecular mechanisms of the functional regulation between HIV-1-associated factors and p53.

## HIV-1-associated factors inhibiting the function of p53

### Nef

**Fig. 1:**
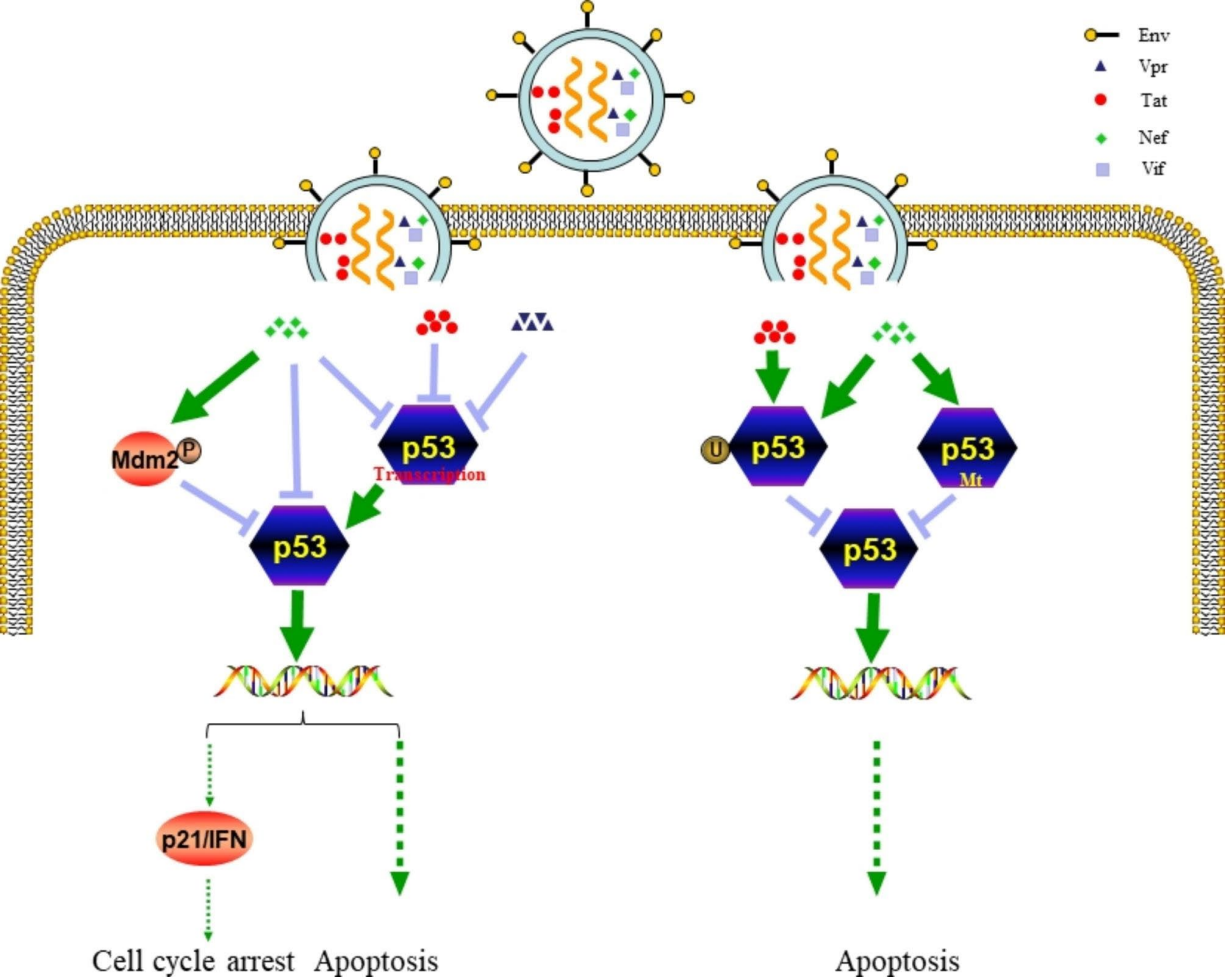
HIV-associated factors inhibit the function of p53. Nef can inhibit p53, impeding cell cycle arrest or apoptosis, dependent or independent of the Mdm2 pathway. Alternatively, nef, tat, and vpr inhibit the function of p53 via inhibiting its transcription. Furthermore, nef and tat inhibit the function of p53 via ubiquitination or mutation

Recently, many studies have shown that HIV-1 nef suppresses the function of p53 by hampering its activity or reducing its expression. Nef may bind to transactivation domain of p53 in N-terminal region. Subsequently, this action blocks the interaction between p53 and transcription coactivators such as transcription factor 31(TAF31) [13]. Alternatively, nef prevents p53 levels from increasing due to stress signals such as ultraviolet (UV) radiation [14]. The inhibition of the p53 apoptotic function by nef is probably due to its ability to reduce the half-life of p53, thereby decreasing its DNA-binding activity and transcriptional sensitization, blocking p53-mediated apoptosis, and extending the survival capability of infected cells [15, 16]. Hepatocellular carcinoma (HCC) is the most usual malignant liver cancer. HIV-1-mediated p53 dysfunction is associated with the survival of CD4^+^ T cells [17]. Hepatitis virus can disrupt the defense of hepatocytes by inducing mutations, silencing *p53*, and accelerating the degradation of p53 directly or indirectly. HIV-1 may impair tumor immune surveillance; therefore, the instability of the liver immune response is further exacerbated when the hepatitis virus is co-infected with HIV-1. Once infected with HIV-1, the virus invades CD4^+^ T cells and induces the AKT-mediated phosphorylation of MDM2 to degrade p53. Therefore, under these dual effects, an early cellular environment conducive to HIV-1 replication is created, thereby maintaining the long-term survival of the infected CD4^+^ T cells [18, 19]. Park et al. proposed that nef is transferred into liver cells through the infected CD4^+^ T cell tract, which significantly increases the production of reactive oxygen species (ROS) in hepatocytes and enhances the replication of ethanol-mediated HCV, thereby accelerating the progression of HCC [20]. Additionally, HIV-1 inhibits the p53/p21/IFN-stimulated gene pathway and type I interferon alpha/beta (IFN-alpha/beta) production by hepatic macrophages [21]. Nef also protects HIV-1-infected cells from p53-induced apoptosis by mediating p53 degradation in the ubiquitin-proteasome pathway, via the cellular E3 ubiquitin ligase E6AP in an Mdm2-independent manner. Using GST pull-down and immunoprecipitation assays, nef was found to interact with E6AP in nef-transfected HEK-293T and HIV-1-infected MOLT3 cells, enhancing the ubiquitination degradation of p53 to inhibit apoptosis. When E6AP was mutated at the C843A site (a major negative E6AP mutant), nef bound to the mutated E6AP without enhancing the ubiquitin-mediated degradation of p53 [22, 23]. In CHME3 cells, the natural host HIV-1 nef also inhibited apoptosis by ubiquitination of p53 mediated by E6AP. Nef lacks an E3 ubiquitin ligase domain and does not use Mdm2 to degrade p53, suggesting it likely uses host cell E3 ubiquitin ligase to ubiquitinate p53 [24, 25]. Further, nef inhibited p53-dependent apoptosis by mutating p53 in immunodeficient virus-associated head and neck squamous cell carcinoma [26] (Fig. 1).

### Tat

HIV-1 tat indirectly degrades p53 via ubiquitination. Although no E6 ubiquitin ligase was found in HIV-1, it promoted proteasome pathway-mediated degradation of p53 by increasing HPV-E6 levels in cervical cancer cells [27]. In vitro, it has been reported that human uterine and cervical cancer cells absorb extracellular bioactive HIV-1 tat, thereby increasing the expression of HPV E6 and decreasing the protein level of the inhibitor p53 [28]. Tat promotes the upregulation of oncogenic HPV-E6 in parallel with a decrease in the level of anti-tumor p53, a potent antagonist of the development and progression of uterine and cervical cancers. Tat downregulates p53 by inhibiting *p53* transcription [29, 30]. The interaction between tat and the coactivator acetyltransferase inhibits p53 Lys-320 acetylation and *p53*-responsive transcription [31]. Tat may also inhibit *p53* transcriptional activity via blocking K320 acetylation. In addition, tat also directly combines *p53* via the *p53* dimerization domain (amino acid residues 341–355), thereby downregulating *p53* promoter activity and inhibiting *p53* transcription and inactivation. As a result, p53 loses its ability to reactivate its downstream target gene, p21WAF1. When cells were treated with the RGD-containing tat protein domain (amino acid residues 65–80), p53 activity was inhibited [32]. Moreover, tat and p53 are co-located to p300/CREB-binding protein-related factors and p300 in the nuclei of IMR-32 human neuroblastoma and PC-12 pheochromocytoma cells. Furthermore, the tat-histone acetyltransferase interaction significantly inhibited *p53* transactivation of the 14-3-3 promoter. Due to tat-histone acetyltransferase binding, p53 acetylation of residue Lys320 by p300/CREB-binding protein-related factors is reduced, as proven by in vivo and in vitro experiments. It was found that tat inhibits the acetylation of p53 through p300 in a dose-dependent manner in vitro. HIV-1-infected Molt-4 cells has shown decreased the acetylation of p53 at Lys320 and Lys370 after UV irradiation. These findings indicate a mechanism by which HIV-1 trans-activators impair the tumor suppressor function of p53 in immune/neuronal-derived cells, thereby profiting to tumor establishment in the period of AIDS. The tumor suppressor p53 induces cell cycle arrest in the G1/S and G2/M phases under genotoxic stress conditions. Individuals affected by AIDS show an increased risk of various tumors, possibly due to the inhibition of p53 Lys-320 acetylation and *p53* DNA transcription resulting from tat-HAT binding, which can enhance harmful mutation acquisition by destroying p53 regulated checkpoint defense [31]. Tat can also inhibit p53-dependent gene expression via its C-terminal domain (amino acid residues 73–86), independent of the coactivator CBP/p300 [33]. In addition, tat inhibits the transactivation of p53 via its transactivation domain. Although tat does not bind directly to p53, this inhibition requires the involvement of the N-terminal domain of *p53*. Therefore, crosstalk between tat and p53 may be related to HIV-1 infection or cell transformation activated by HIV-1 replication. p53 is inactivated by binding indirectly to tat in HIV-1 infected cells. Subsequently, p53 is inactivated and loses the ability to reactivate its downstream target gene p21/waf1, a well-known CDK inhibitor (CKI) that can cause cell cycle arrest upon DNA damage [34, 35] (Fig. 1).

### Vpr

HIV-1 Vpr regulates DNA replication and cell-cycle progression. Vpr-binding protein (VprBP) inhibits p53 transcription by regulating histone acetylation, thereby regulating cell viability and apoptotic pathways. *p53* recruits VprBP to its responsive promoters, and this action inhibits *p53* transactivation in the absence of stress stimuli [36]. It stably binds to the nucleosome by recognizing the unacetylated H3 tail to maintain the target promoter in an inactive state. The prerequisite for VprBP tethering and serving as an inhibitor of p53 target genes is the promoter-localized deacetylation of H3 tail. Knocking down VprBP leads to the activation of *p53* target genes, resulting in an increased cell apoptosis induced by DNA damage. Furthermore, phosphorylating VprBP at serine 895 weakens its ability to bind to the H3 tail and inhibits *p53* trans-activation. Therefore, a novel role of VprBP in regulating p53 signaling was revealed, as well as the molecular mechanisms of cancer development associated with VprBP dysregulation. Recruitment of VprBP to the *p53* promoter region fades p53-dependent transcription. This may occur via the interaction of VprBP with histone H3 tails and the inhibition of acetylation in the promoter region. HDAC1-mediated H3 tail deacetylation helps the stable localization of VprBP on the *p53* target promoter [37–39]. Therefore, VprBP regulate cell viability and p53 target gene expression (Fig. 1).

## HIV-1-related factors activating the function of p53

### Tat

**Fig. 2:**
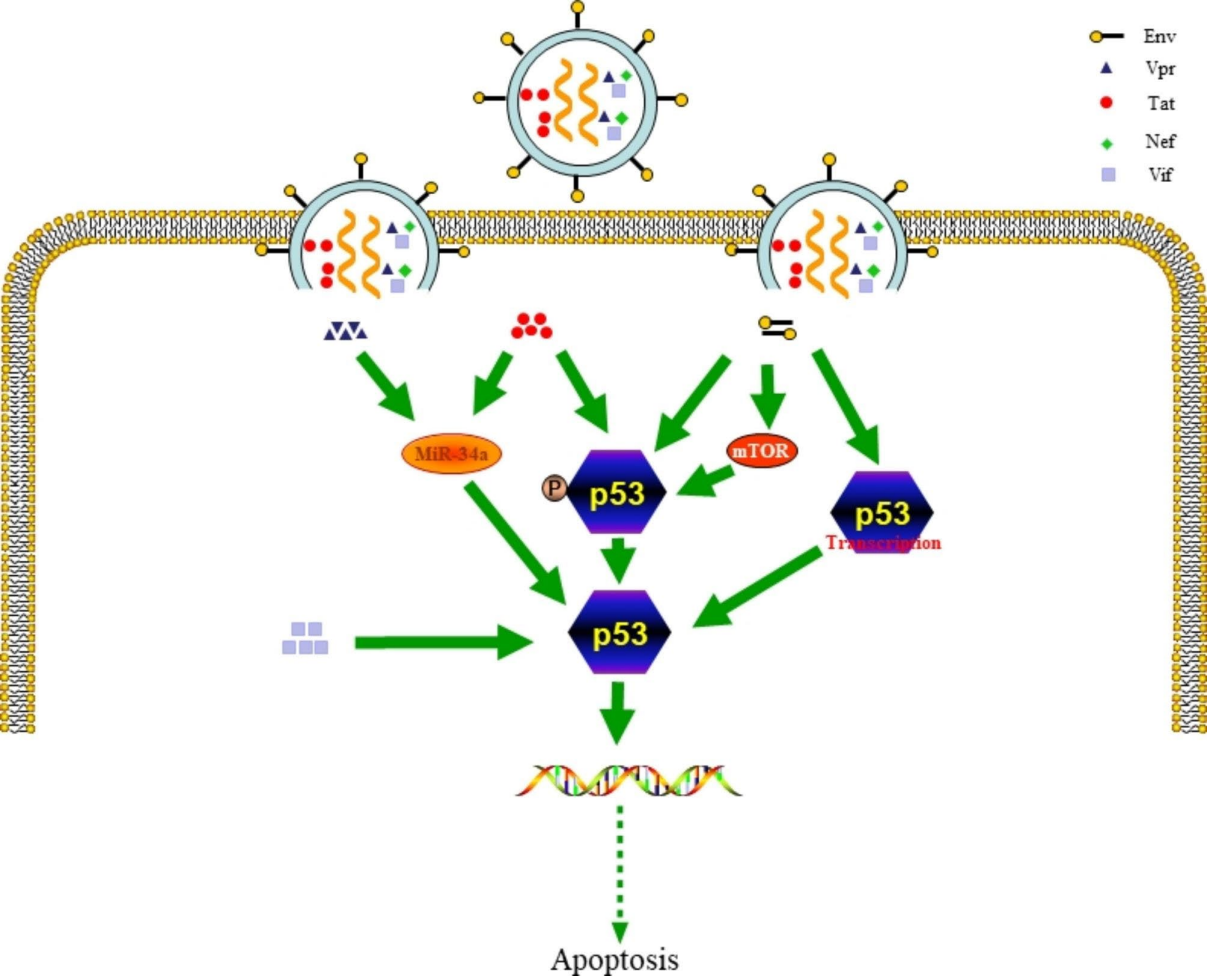
HIV-1-related factors activate p53. Vpr and tat enhance the function of p53 by upregulating miR-34a. In addition, tat also enhances p53 by phosphorylation. Furthermore, env enhances the function of p53 through the mTOR or non-mTOR pathways, or by promoting the transcription of p53. Vif promotes the function of p53

Tat inhibits *p53* transcription or promotes p53 ubiquitination to reduce p53 expression; however, it can also trigger p53 activation. Tat can upregulate miR-34a in neurons to initiate p53 activation and indirectly promote p53 accumulation by phosphorylating p53 serine residues 15 and 46 [40]. Tat induces p53 phosphorylation in the neural cells SH-SY5Y, and this induction leads to neurite retraction. Without p53, tat fails to induce the retraction of dendrite or activate pro-apoptotic proteins such as Bax. Thus, tat promotes neuronal degeneration by activating the p53 pathway. This information is relevant for developing therapeutics that impact these pathways to protect the central nervous system (CNS) neurons and prevent HIV-associated dementia (HAD) [41, 42]. 30 kDa HIV-1 tat interacting protein (TIP30), also known as CC3 or HTATIP2, is a tumor suppressor that functions as an angiogenesis inhibitor [43]. TIP30 intercepts the nuclear import of the mRNA-binding protein HuR, thus promoting HuR cytoplasmic accumulation via combining with the β protein of importation. The accumulation of the mRNA-binding protein HuR in the cytoplasm upregulates the transcription factor *p53*. In addition to the post-transcriptional mechanism of increasing p53 levels through TIP30, it has been proposed that TIP30 regulates the levels of p53 by direct binding. In order to explore the probability of the direct interaction between p53 and TIP30, the researchers selected three functional regions of p53 to study their interactions using GST pulling and surface plasmon resonance techniques. These results indicated that TIP30 combines with p53 in DNA-binding and C-terminal domains to upregulate p53 [44] (Fig. 2).

### Env

The mechanisms by which HIV-1 infection increases cellular senescence are diverse among which one critical signaling pathway is that of p53 activation. HIV-1-env activate p53 pathway to induce cell apoptosis. Syncytia causing by the fusion of env and CD4^+^ T cells leads to mammalian target of rapamycin (mTOR)- mediated nuclear uptake of p53 phosphorylation (S15 and S46), p53-dependent Bax upregulation, and mitochondrial death pathways activation, which cause cell apoptosis [42, 45].Dominant negative *p53* transfection inhibits apoptosis. The phosphorylation of p53 (S15 and S46 residues) in lymph node biopsies and peripheral blood mononuclear cells from HIV-1 infected patients are correlated with the viral load. Microarrays show that about 85% of Env transcription are mediated by p53 inhibitor pifithrin-α. Thus, HIV-1 Env relies on the p53 pathway to initiate pro-apoptotic signaling. It has been observed that env expression and diabetes synergistically induce the activation of p53 in Podocytes to trigger senescence and apoptosis signaling cascades [46]. Studies on HIV-1-infected primary macrophages have shown that env regulates apoptosis by inducing p53 phosphorylation [47]. A microarray study reveals that env induces rapid upregulation of type-I interferon-dependent *p53* mRNA in human primary CD4^+^ T cells and subsequently induces apoptosis [48]. The envelope protein Gp120 of HIV-1 is involved in the activation of the p53-mediated apoptosis pathway. In cultured neurons, Gp120 stimulates p53 activity and induces the expression of the p53 pro-apoptotic target Apaf-1 [49]. Gp120 also promoted the phosphorylation of p53 and expression of p53 transcriptional targets. In a model of HIV-associated neurocognitive disorders (HAND) in vitro, soluble Gp120 (200 pM) was added to a mixed cortical culture containing mouse neurons, leading to p53 of strongly activation. Moreover, in mixed cortical cultures of p53-deficient mice exposed to Gp120, neurons are antagonistic against Gp120-induced apoptosis, suggesting that the pathway of p53 is activated during Gp120-induced neuronal injury [50]. In addition, the brain tissue analysis from HIV-1-infected patients with severe dementia showed that p53 is activated in both neuronal and non-neuronal cells [51] (Fig. 2).

### Vif and vpr

p53 is stabilized and activated by Vif. This effect induces G2 phase arrest in infected cells, thereby supporting HIV-1 replication [52]. Vpr triggered the activation of p53 by upregulating miR-34a in neurons [53] (Fig. 2).

## HIV-associated factors that can be inhibited by p53

### Tat

p53 inhibits the transcription or activity of tat, thereby inhibiting HIV-1 replication in vivo. Therefore, it is advantageous for HIV-1 to maintain low p53 levels to achieve optimal transcription and replication. The p53 dimerization domain (amino acid residues 341–355) directly binds to the *tat* promoter and represses its transcription [34]. Recently, A report reviewed that in colorectal cancer p53 induces the expression of double-stranded RNA (dsRNA)-dependent protein kinase R (PKR) and subsequently inactivates tat through phosphorylation. *p53* silencing can strengthen the replication of HIV-1 in infected cells, whereas the overexpression of p53 reduces tat activity, which is reversed by knocking out PKR. Further studies have revealed that PKR interacts directly with tat, and then phosphorylating its first exon in five Ser/Thr residues (S68, S62, S46, T40, and T23), thereby repressing tat-mediated proviral transcription. In patients infected with HIV-1 strains, p53 is essential for the inhibition of HIV-1 replication through tat inactivation. In the HIV-1 strains circulating among the United States and Europe, five Ser/Thr residues are highly conserved and subject to host limitations [54–56]. The small chemical molecule 9-aminoacridine (9AA), a p53 activator, reactivates p53-p21/Waf1 and inhibits the replication and transcription of HIV-1. With the interaction between p53 and HIV-1 being critical in HIV-1 research, the mutually antagonistic relation of p53 and tat is of specific importance. This interaction leads to the repression of tat transcription via p53 and the downregulation tat-mediated p53-dependent transcription [32].

### HIV-1 LTR

**Fig. 3:**
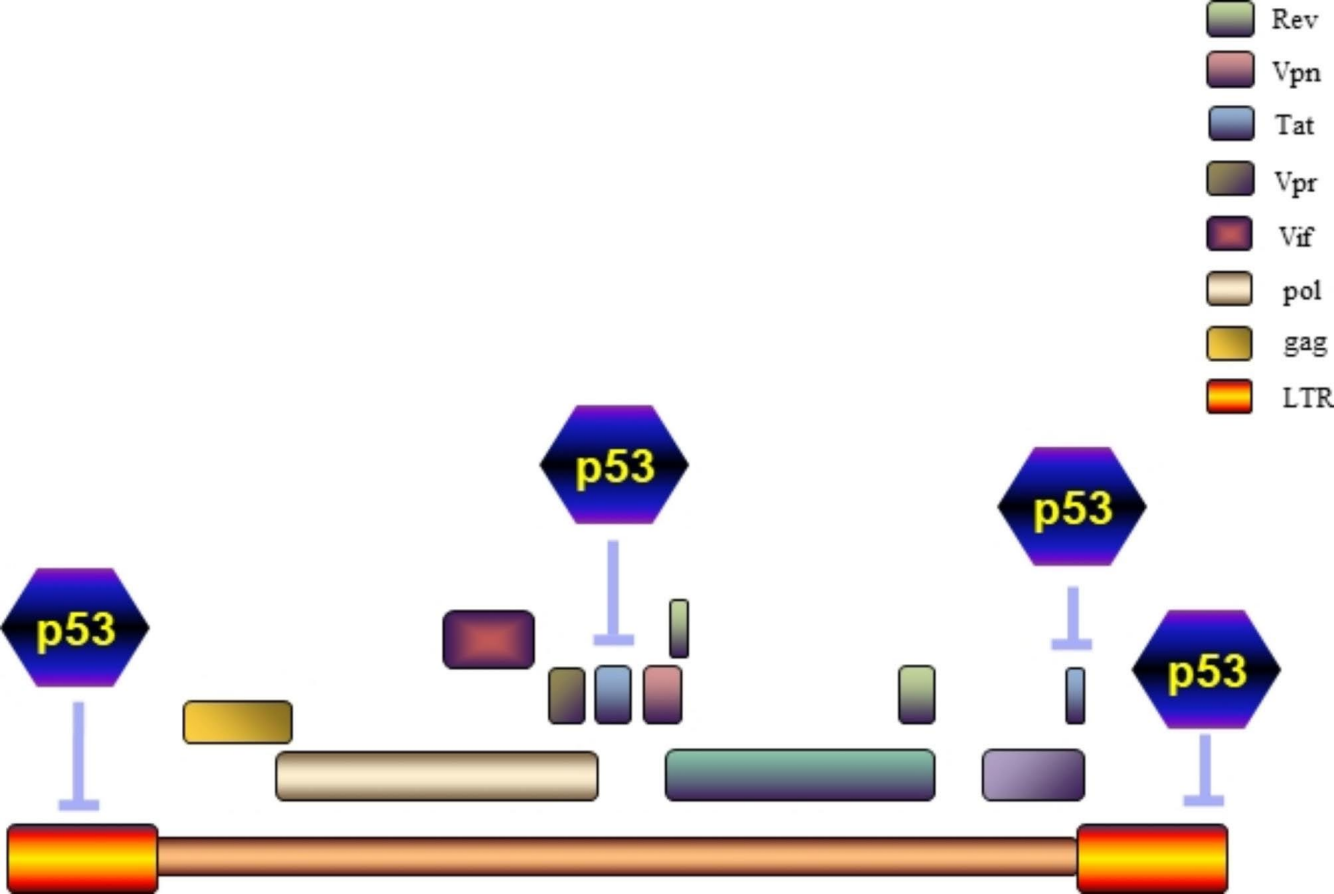
p53 inhibits HIV-associated factors. p53 inhibits HIV-1 replication by impeding the transcription or activity of tat, or by inhibiting the transcriptional regulation of LTR.

p53 is negatively involved in transcriptional regulation of HIV-1 long terminal repeats (LTR). However, the mechanisms responsible for this negative effect are not yet clear. p53 was found to inhibit the LTR promoter activity and the transcription of HIV-1 provirus genome [57]. Other studies have shown that overexpressing wild-type p53 (wt-p53) can block the phosphorylation of Ser2 in the RNA polymerase II carboxy-terminal domain. Inhibition of Ser2 phosphorylation blocks transcriptional elongation in the LTR, thus significantly reducing the replication of HIV-1 in primary microglia and astrocytes. This effect was mitigated by depleting p53 in cells using siRNA- mediated silencing of *p53*. Inhibition of p53 expression leads to an increase in pol II levels associated with the HIV-1 genome sequences, transcripts elongation, and amplified viral replication [58, 59]. Other studies have also found that the N-terminal acidic structural domain of p53 can combine directly with TATA-box binding protein subunit of the universal transcription factor TFIID. In a co-transfection assay system, wt-p53 restrains LTR-directed chloramphenicol acetyltransferase activity. Significantly, in vitro transcription assays also demonstrates the effect of wt-p53 on LTR [60] (Fig. 3).

## Conclusions and prospectives

The co-regulatory roles of HIV-1-related factors and the p53 protein during infection are being explored. It is known that HIV-1-related factors are associated with various host proteins. In this article, we review the regulatory roles of HIV-1-related factors and p53 in cellular processes and the corresponding molecular mechanisms. The current standpoints believe that in the microenvironment of tumors, HIV-1 infection reduces the expression of p53 and accelerates the occurrence and development of tumors [61]. In non-tumor microenvironments, HIV-1 infection promotes the expression of p53, accelerating cell aging and death [62]. This article summarizes that Nef, tat, and vpr inhibits apoptosis and cell cycle arrest by directly or indirectly reducing p53 expression. Tat, env, vif, and vpr can promote p53 expression and the apoptosis of target cells. P53 can also inhibit the expression of HIV-1 by inhibiting the transcription of Tat or HIV-1 L, so p53 may be a novel therapeutic target for the inhibiting of HIV-1 gene expression and replication as well as AIDS treatment [63].

Currently, research on HIV-1 and p53 co-occurrence is scattered, and information on the mechanisms of action and effects is limited. For example, in HIV-1-infected HUT78 T cells, studies only found that p53 expression levels were reduced, but the exact mechanism was unclear [64]. After HIV-1 virus infection, type I interferon (IFN-α/β) activate p53 expression in human immune cells. Thus, HIV-1 may impair tumor immune surveillance [19]. HIV-1 infection leads to immune cell death through the induction of p53-dependent apoptosis [65]. In addition, p53 may affect HIV-1 reverse transcriptase function [66]. p53 strongly affects the genetic expression of microglia and transcriptional responses to HIV-1 Gp120. However, the detailed cellular mechanism remains to be determined [67]. The balance among p53 isoforms regulates HIV-1 infection efficiency in macrophages. Indeed, increased p53 levels led to strong restriction of the early replication steps of HIV-1 by SAMHD1 activity [68]. In HIV-1 infected cells, treatment with 9AA also reactivates the p53 pathway [69]. Therefore, the relationship between HIV-1-related factors and p53, and the related molecular mechanisms, warrants further study.

Therefore, it is important to further study the regulation, relationship, and corresponding molecular mechanisms between HIV-1-related factors and the p53 protein, which are conducive to an in-depth understanding of the nature and treatment of diseases.

## Data Availability

Not applicable.
